# Recent advances in the use of *Trichoderma*-containing multicomponent microbial inoculants for pathogen control and plant growth promotion

**DOI:** 10.1007/s11274-024-03965-5

**Published:** 2024-04-13

**Authors:** László Kredics, Rita Büchner, Dóra Balázs, Henrietta Allaga, Orsolya Kedves, Gordana Racić, András Varga, Viktor Dávid Nagy, Csaba Vágvölgyi, György Sipos

**Affiliations:** 1 Department of Microbiology, Faculty of Science and Informatics, University of Szeged, Közép fasor 52, Szeged, 6726 Hungary; 2 Faculty of Ecological Agriculture, Educons University, Vojvode Putnika 87, Sremska Kamenica, 21208 Serbia; 3 Functional Genomics and Bioinformatics Group, Institute of Forest and Natural Resource Management, Faculty of Forestry, University of Sopron, Sopron, 9400 Hungary

**Keywords:** *Trichoderma*, Biocontrol, Plant growth promotion, Microbial inoculant

## Abstract

Chemical pesticides and fertilizers are used in agricultural production worldwide to prevent damage from plant pathogenic microorganisms, insects, and nematodes, to minimize crop losses and to preserve crop quality. However, the use of chemical pesticides and fertilizers can severely pollute soil, water, and air, posing risks to the environment and human health. Consequently, developing new, alternative, environment-friendly microbial soil treatment interventions for plant protection and crop yield increase has become indispensable. Members of the filamentous fungal genus *Trichoderma* (Ascomycota, Sordariomycetes, Hypocreales) have long been known as efficient antagonists of plant pathogenic microorganisms based on various beneficial traits and abilities of these fungi. This minireview aims to discuss the advances in the field of *Trichoderma*-containing multicomponent microbiological inoculants based on recent experimental updates. *Trichoderma* strains can be combined with each other, with other fungi and/or with beneficial bacteria. The development and field performance of such inoculants will be addressed, focusing on the complementarity, synergy, and compatibility of their microbial components.

## Introduction

The genus *Trichoderma*, belonging to the Hypocreaceae family (Hypocreales, Sordariomycetes, Ascomycota), is a highly studied group of filamentous fungi known for its numerous beneficial traits, particularly in agriculture as key components of microbiological inoculants. According to the most recent taxonomic concept, more than 400 described species of the genus are divided into 8 main clades (Cai and Druzhinina 2021).

Although some *Trichoderma* species undergo sexual reproduction, the majority are better adapted to an asexual life cycle, leading to significant variation in chromosome number and size among strains, even without meiosis (Harman et al. 1993; Kistler and Miao 1992). Events that modify the asexual genetic pool, such as parasexual recombination and mutations, allow cells within the same thallus to exhibit significant genetic diversity. Due to their high genetic plasticity, *Trichoderma* species form a rapidly adapting and evolving group of filamentous fungi (Kubicek and Harman 1998).

Members of the genus *Trichoderma* are widely distributed (Kubiak et al. 2023). The genus includes soil-dwelling microorganisms found in almost all types of soil worldwide and are often seen as plant symbionts, saprotrophs and mycoparasites (Alfiky and Weisskopf 2021; Kubiak et al. 2023). *Trichoderma* species possess advanced endosymbiotic capabilities, enabling them to support plant hosts, adapt competitively to microbial environments, and colonize a wide range of soil ecosystems (Harman et al. 2004). In *Trichoderma*, such essential, highly adaptive functional traits combine to form a coherently evolved group of fungi ideally suited to restore a diseased plant environment, thus capable of promoting eco-friendly, sustainable agricultural applications. Several *Trichoderma* species have the potential to protect against plant pathogenic moulds through competition for space and nutrients, the production of secondary metabolites, and mycoparasitism (Kredics et al. 2021).

Some *Trichoderma* species also cause significant economic losses in mushroom cultivation such as *Trichoderma aggressivum* f. *aggressivum* (Hatvani et al. 2017) or members of the *Trichoderma harzianum* species complex (THSC) (Allaga et al. 2021), while other *Trichoderma* species are known as eventual plant pathogens, such as *T. afroharzianum* (Pfordt et al. 2020), or as opportunistic human pathogens causing trichodermosis, such as *Trichoderma longibrachiatum* (Naeimi et al. 2022). To minimize the risks associated with *Trichoderma* application, careful consideration and selective use of certain taxa in agriculture are recommended.

In this review we focus on the application of *Trichoderma* in combination with other microorganisms for the formation of multicomponent microbial inoculants for pathogen control and plant growth promotion.

## Beneficial traits of *Trichoderma* species

*Trichoderma* species exhibit diverse saprotrophic and mycoparasitic abilities, equipping them effectively for rhizosphere colonization and interaction within plant tissues. They rapidly adapt to their interactive environment by dynamically remodeling chitin-related structures of their cell surface (Kappel et al. 2020) and producing a variety of compounds that bolster the plant defense responses and enhance plant vigour by activating beneficial biochemical pathways.

### Antifungal and antinematode traits

*Trichoderma* species exhibit a wide range of antifungal properties effective against fungal plant pathogens (Druzhinina et al. 2011; Chen et al. 2023). They secrete hydrolytic enzymes including chitinases, glucanases, lipases, and endo- and exopeptidases targeting various cell wall components of the interacting fungal partners. Besides enzymatic effectors, they can externally deliver potent bioactive substances, including a broad spectrum of secondary metabolites (SMs) inhibiting fungal competitors or acting biofungicidal on susceptible, noncomplementary fungal cells (Kubiak et al. 2023).

The production of cell wall degrading enzymes (CWDEs) makes *Trichoderma* species pioneers in degrading chitin, lignin, cellulose and hemicellulose due to the secretion of extracellular enzymes. The activity of these enzymes is widely studied through the expression of genes responsible for their synthesis (Tyśkiewicz et al. 2022). Cellulolytic enzymes of *Trichoderma*, mainly exo- and endo-β-1,4-glucanases, and β-glucosidases are responsible for the hydrolysis of β-1,4-D-glycosidic bonds, thus creating advantage for the species of *Trichoderma* to utilize cellulose as a carbon source when colonizing different ecological niches where this polysaccharide is available (Strakowska et al. 2014). *T. atroviride* has been studied for β-1,3-glucanase activity as one of the biocontrol mechanisms to parasitize the survival structures of plant pathogens, such as sclerotia (Kaur et al. 2005).

A number of bioactive metabolites of *Trichoderma* spp. are crucial for their antifungal activities. Terpenes inhibit fungal growth (Adamczyk et al. 2023) and pyrones interfere with spore germination and mycelial growth (Liu et al. 2022a; Degani and Gordan 2022). Others, such as gliotoxin and gliovirin, originally identified as antibiotic substances in some *Trichoderma* species, can induce oxidative stress and disrupt the intracellular redox homeostasis in fungal cells (Scharf et al. 2016). Peptaibols, representing short-chain linear polypeptides, secreted as a mixture of isoforms carry a membrane disrupting ability to form pores in lipid membranes (Tieleman et al. 1999; Vey et al. 2001). So far hundreds of sequences have been identified and their potent antifungal impact confirmed at concentrations where negative effects on plants were still not detected (Szekeres et al. 2005; Marik et al. 2019). *Trichoderma* genomes are enriched in polyketide synthase (PKS) genes (Baker et al. 2012) representing one of the main fungal secondary metabolite pathways whose products in general may inhibit fungal growth. *Trichoderma* cells also produce volatile organic compounds, representing diverse chemical structures such as ketones, terpenes, lactones and alcoholic substances which may also impose an inhibitory or mycotoxic effect on the susceptible fungal partners (Korpi et al. 2009; Siddique et al. 2012; Kong et al. 2022).

Strong biocontrol activity against plant parasitic nematodes (PPNs) was also found by *Trichoderma* species. Modes of action include both destruction of nematode cuticule by the production of hydrolytic enzymes and volatile and non-volatile metabolites, as well as direct parasitism of larvae and eggs. Investigations carried out so far, showing interwinding combination of different mechanisms of *Trichoderma* species to control PPNs (Poveda et al. 2020; De Oliveira et al. 2021; Almeida et al. 2022; Tiwari et al. 2021; Baazeem et al. 2021) are important steps in designing and incorporating these agents into integrated pest management strategies.

### Plant stimulating traits

*Trichoderma* species can colonize the roots and trigger induced systemic resistance through jasmonic acid and ethylene or induce systemic acquired resistance by the salicylic acid pathway (Ab Rahman et al. 2018), which lays the foundation for protection not only against plant pathogens, but also against the effect of various stress factors. Through the accumulation of lipid peroxides in seedlings under stress conditions, *T. harzianum* could provide early protection against oxidative damages (Mastouri et al. 2010). Based on the researches of the past years, the application of *Trichoderma* species and their produced metabolites could alleviate biotic and abiotic stresses in plants (Abeed et al. 2022; Rawal et al. 2022; Contreras-Cornejo et al. 2014; Zhao and Zhang 2015; Oljira et al. 2020; Brotman et al. 2013).

In addition to conditioning for different adverse environmental effects, *Trichoderma* species also affect the development and growth of plants. The effect of *Trichoderma* strains on tomato (Macías-Rodríguez et al. 2018; Wang et al. 2021; Sehim et al. 2023), cucumber (Liu et al. 2021; Yedidia et al. 2001), cotton (Silva et al. 2022), as well as medicinal (Huong et al. 2022) and ornamental plants (Andrzejak and Janowska 2022) has already been investigated in numerous studies. As a result of the *Trichoderma* treatments, an improvement in stem, leaf and root development, as well as increased amount of photosynthetic pigments was observed in several cases.

## Expectations from *Trichoderma* bioinoculants: the Super*Trichoderma* concept

Here we introduce the concept of „Super*Trichoderma*” by listing the desired traits and expectations that should be met by a *Trichoderma*-based biocontrol product. An ideal biocontrol *Trichoderma* strain should grow fast, produce large amounts of conidia, have excellent antagonistic abilities by efficient competition and the capacities of antibiosis and mycoparasitism against a wide variety of plant pathogenic fungi. In addition, a flawless bioeffector strain must be able to promote plant growth and induce systemic resistance, be rhizosphere competent with many crops, efficiently degrade stem residues, and be compatible with other means of control (including chemicals by pesticide-polyresistance). Finally, the selected biocontrol candidate should also detoxify the soil, and should not be harmful or pathogenic to crop plants, cultivated mushrooms, farm animals and humans. This “Super*Trichoderma*” concept is, of course, rather idealistic, as achieving all the above goals with a single *Trichoderma* strain is probably impossible. Furthermore, using single strains as inoculants may also result in an inconsistent field performance because of varying biotic and abiotic environmental conditions. However, using a combination of microorganisms in the form of synthetic communities (SynCom) to mimic the structure and function of microbial communities and to realize synergistic plant-beneficial effects of their components is an alternative approach for the development of efficient inoculants. *Trichoderma* strains can be combined with other *Trichoderma* partners, other beneficial fungi (e.g., *Coniothyrium*, *Glomus*, *Chaetomium* and *Beauveria* spp.), and/or beneficial prokaryotes (e.g., *Bacillus* and *Pseudomonas* spp. or nitrogen-fixing bacteria).

## Selection of *Trichoderma* strains for microbial inoculants

The selection of a biocontrol *Trichoderma* strain should be tailored to the specific disease challenges and crop conditions in each agricultural system. Additionally, proper testing and field trials are necessary to validate the performance of any biocontrol candidate under greenhouse or field conditions (Hyder et al. 2017).

The most frequently used in vitro methods for the selection of *Trichoderma* strains are classical microbiological methods. *Trichoderma* strains are usually isolated from the rhizosphere of healthy plants and soil, into potato dextrose agar or *Trichoderma*-selective medium plates using serial dilution technique (Herrera-Jiménez et al. 2018; Saxena et al. 2015; Singla 2019). The reliable identification of the isolated *Trichoderma* strains requires the sequence analysis of three DNA barcodes (ITS, *tef1*, and *rpb2*), which is supported by online tools available at www.trichokey.info (Cai and Druzhinina 2021). In order to evaluate the antagonistic effect of *Trichoderma* strains, the confrontation of antagonistic isolates and pathogens is usually carried out using a double culture technique (Chen et al. 2019; Degani et al. 2023; Nagy et al. 2023). The ability of *Trichoderma* strains to produce extracellular enzymes such as protease, phosphatase, cellulase, chitinase and glucanase is most often assessed by colorimetric methods, using dinitrosalicylic acid (DNS) or chromogenic substrates to determine the amount of enzyme production (Go et al. 2019; Mustafa et al. 2020; Xue et al. 2021). The most common method to evaluate auxin (indole-acetic acid) synthesis by *Trichoderma* strains is the Salkowski test (Abdenaceur et al. 2022; Bader et al. 2020; López 2023; Nagy et al. 2023). Siderophore production is commonly analyzed by the Chrome Azurol assay (Hussein and Jin 2015; Karličić et al. 2021; Zhang et al. 2013). The phosphorus solubilization analysis can be performed by the National Botanical Research Institute Phosphate method (Bononi et al. 2020; Gezgin et al. 2020). In addition, secondary metabolites that stimulate plant growth, induce plant defense, inhibit the growth of plant pathogenic fungi and bacteria, or help the plant to overcome abiotic stress are commonly qualitatively and quantitatively analyzed by gas chromatography or high performance liquid chromatography - mass spectrometry (Wu et al. 2017; Zhou et al. 2021).

To select *Trichoderma* strains for microbial inoculants using omics-directed screening involves utilizing various high-throughput molecular biology techniques and bioinformatics to identify strains with beneficial traits at the genetic and molecular levels. Omics-directed screening allows for a more systematic and data-driven approach to select *Trichoderma* strains with specific traits. It leverages the power of genomics, transcriptomics, proteomics, and metabolomics to identify promising candidates for improving soil health, plant growth, and disease management (Dutta et al. 2023; Mukherjee et al. 2013). The integration of metabolomics data with genomics, transcriptomics, and proteomics data enables to gain a comprehensive understanding of biological processes (Lorito et al. 2010; Zhang et al. 2016; Chen et al. 2023).

## Multicomponent inoculants containing *Trichoderma*

### Combination of multiple *Trichoderma* strains

While the beneficial effects of the application of single *Trichoderma* strains on plants are widely known in the literature (Lorito and Woo 2015; López-Bucio et al. 2015), the co-application of multiple *Trichoderma* strains has come to the foreground only in the recent years (Table 1). The combined use of multiple species may provide further support in plant development and protection against phytopathogenic microorganisms through the activation of silent gene clusters and secondary metabolite production (Netzker et al. 2015; Knowles et al. 2022). They can more easily facilitate adaptation and form a stable, beneficial microbial composition in the soil, and may also provide a wider support for plant development and protection against pathogens. One of the most important issues of the co-application of multiple *Trichoderma* strains is the optimization of the applied ratios and the methods of inoculation (Liu et al. 2022a).

**Table 1:** Combination of *Trichoderma* strains and other microorganisms for potential agricultural application

Combination	Case	Type of study	Positive effect	Reference
Multiple *Trichoderma*				
*T. asperellum* and *Trichoderma* sp.	Fusarium wilt in banana (*Fusarium oxysporum* f. sp. *cubense*)	in vitro and greenhouse	inhibiton of *Fusarium*, 100% reduction of Fusarium wilt disease, increase in plant growth parameters up to 250%, increase in IAA production and phosphate solubilisation	Thangavelu and Gopi 2015
*T. asperellum, T. harzianum* and *T. virens*	*Ganoderma boninense* in oil palm	nursery trial	reduction in foliar disease symptoms (by 83.03%) and bole damages (by 89.16%), increase in plant height and girth, dried shoot and root tissue weight	Musa et al. 2018
*T. tomentosum* and *T. harzianum*	tryptophan supplementation in *Zea mays*	microcosm	increase in plant height, root length, leaf area, and dry weights in maize	Herrera-Jiménez et al. 2018
*T. atroviride* and *T. citrinoviride*	*Brassica chinensis* growth promotion	in vitro and greenhouse	elevation of IAA and iron-chelating siderophore content, improved germination parameters and seedling growth	Chen et al. 2021
*T. asperelloides, T. asperellum* and *T. harzianum*	*Fusarium oxysporum*, seed germination promotion in cucumber	in vitro, artificial climate indoor cultivation, and field	increase in amino acid and γ-aminobutyric acid levels, increased plant height and stem diameter	Hao et al. 2022
Two *T. asperellum* isolates	Fusarium root and stem rot in cucumber plants *(Fusarium oxysporum* f. sp. *radices-cucumerinum)*	greenhouse	reduction in reactive oxygen species accumulation and activation of antioxidant enzymes, strong mycoparasitic effect in the case of application of multiple *Trichoderma* strains	El-Komy et al. 2022
*T. viride* and *T. harzianum*	*Fusarium oxysporum* in cherry tomatoes	greenhouse	reduction in plant pathogen growth and more favourable effect on plant development	Awad-Allah et al. 2022
*T. koningiopsis* and *T. asperellum*	paddy crop under salt stress conditions	greenhouse	support of plant growth (stimulation of root and shoots growth, nutrient uptake, production of photosynthetic pigment, triggered activity of antioxidant enzymes), mitigation of the impact of environmental stress	Anshu et al. 2022
*Trichoderma* with other fungi				
*Trichoderma harzianum* and *Glomus intraradices*	Fusarium crown and root rot in tomato cultivation	field	inhibition of disease incidence and severity, increased fruit size	Datnoff et al. 1995
*Trichoderma* sp. and AMF	competing with *Glomus* spp. on pea roots	in vitro	mycoparasitic activity against AMF	Rousseau et al. 1996
*T. harzianum* and *Glomus versiforme*	powdery mildew (*Erysiphe flexuosa*) in cowpea	greenhouse	enhancement of cowpea seedlings’ resistance to powdery mildew and growth promotion (increase in shooth dry weight)	Omomowo et al. 2018
*T. harzianum* and AMF	Fusarium wilt in bananas	greenhouse	reduction of Fusarium wilt infection, increase in plant height, pseudostem diameter and root weight	Castillo et al. 2019
*T. asperellum* and *Beauveria bassiana*	Asian corn borer on maize	in vitro and greenhouse	suppression of the Asian corn borer’s immune response resulting in 98.3% mortality, induction of plant resistance	Batool et al. 2020
*Trichoderma* spp. and mycorrhizal fungi	apple replant disease	tree nursery	induction of growth in root system and aerial parts of trees in replant soil	Zydlik et al. 2021
*T. viride* and dark septate endophytes	drought stress of *Astragalus mongholicus*	growth chamber	synergism in driving the rhizosphere microbiome and plant adaptive responses towards drought resistance, increase in plant biomass, plant height and root surface area	He et al. 2022
*Trichoderma* with bacteria				
*T. harzianum*, *B. subtilis*	biocontrol of the anise pathogen *Puccinia pimpinellae*	field	moderate decrease in disease incidence and severity in treatments due to pathogen inhibition by chitinase production	Saber et al. 2009
*Trichoderma* sp., *T. atroviride*, *T. virens*, *P. chlororaphis*, *P. pseudoalcaligenes*	white rot caused by *Rosellinia necatrix* in avocado plants	greenhouse	significant reduction in disease level and onset of symptoms due to antibiotic production by bacteria and good competency of *Trichoderma* strains against the pathogen	Ruano-Rosa et al. 2014
*T. harzianum*, *Azospirillum brasilense*	grain treatments of wheat and corn	pot and field	positive effect on seedling growth and yield parameters, protection against *Fusarium* sp., 50% disease incidence reduction, 53% shoot height increase	El-Katatny and Idres 2014
*T. harzianum*, *Pseudomonas fluorescens*	tomato seedlings treated with colonized neem cake	pot	enhancement of fruit yield and improvement in plant growth (root length increase by 91–193%, shoot length increase by 37-59.5%)	Tomer et al. 2015
*T. harzianum*, *B. subtilis*	seed coating formulation for vegetable seeds	greenhouse	reduction in disease incidence, increase in root length (35.5%), fresh weight of roots (54.5%), shoot length (40.8%), yield increase in turmeric crops	Kumar et al. 2015
*T. viride, Azotobacter chroococcum*	biofilm formation on chickpea	greenhouse	increased shoot length (20%), root length (12%), root volume (27%), protein content in leaves (30%) and roots (46%), increased activity of plant enzymes	Velmourougane et al. 2017
*T. asperellum*, *B. amyloliquefaciens*	LC–MS/MS examination of the metabolites in co-culture	in vitro	increase in amino acid and biocontrol metabolite production in co-cultures, 47.86% higher antimicrobial activity against *B. cinerea*	Wu et al. 2018
*T. harzianum*, *P. fluorescens*	seed treatment and foliar spray application of the agents on maize against *Rhizoctonia solani*	greenhouse	induction of systemic resistance, plant disease incidence decreased from 89–37.7% in co-culture treated plants	Madhavi et al. 2018
*T. viride, Rhizobium* sp.	seed biopriming of French bean	field	field emergence elevated from 78.51–91.48%, pod yield increased from 25.93 g to 36.28 g, disease incidence reduced from 12.8–3.71%	Negi et al. 2019
*T. asperellum*, *B. amyloliquefaciens*	wheat seed treatment with co-culture	in vitro and greenhouse	protection against plant pathogens and improvement in plant growth, reduced disease incidence of *F. gramineum*, during germination tests	Karuppiah et al. 2019a
*T. asperellum*, *B. amyloliquefaciens*	co-culture of the agents and treatment of maize seeds	in vitro and greenhouse	increase in plant growth (shoot, root and seedling length) and disease resistance, reduction of *F. graminearum* growth by 71.5–80.1%	Karuppiah et al. 2019b
*T. harzianum*, *B. subtilis*	investigation of the biocontrol potential against *Streptomyces* spp.	field	decrease in disease incidence (37.7–40.1%), increase in yield	Wang et al. 2019
*T. asperellum*, *B. amyloliquefaciens*	spray treatment and growth medium application against tomato bacterial spot caused by *Xanthomonas perforans*	growth chamber	potential for infection control, decrease in disease severity (29,5–31.5%)	Chien and Huang 2020
*T. virens*, *B. velezensis*	effects of the two agents against *Ralstonia solanacearum*	greenhouse	reduction in disease incidence by 50%, increase in plant heights, weights, SPAD values, and defensive enzyme activities in treated plants	Zhou et al. 2021
*T. atroviride*, *B. subtilis*	coating against *Fusarium graminearum* infection in cucumber	in vitro	reduction in pathogen growth (66.2%) and mycotoxin production	Liu 2022c
*T. atroviride*, *B. subtilis*	Botryosphaeria dieback and black-foot disease of grapevine	nursery	efficient reduction in disease incidence (from 18.75–33.75% to 2.5–7.5%)	Leal et al. 2023
*T. harzianum*, *B. subtilis*	seed coating of maize and field application against *Tanymecus dilaticollis* in maize	field	reduction in crop damage (from 16–19% to 6–11%), increase in plant growth and yield	Petcu et al. 2023
*Trichoderma* with multiple microorganisms				
*T. asperellum, Pseudomonas fluorescens, Rhizobium* sp.	treatment of seeds of chickpea and bean	greenhouse and field	improvement in seed germination (up to 80%) and plant growth (100% increase in dry biomass)	Yadav et al. 2013
*Trichoderma asperellum*, *T. atrobrunneum*, *Streptomyces albus*, *Azotobacter vinelandii*	composite soil bioinoculant in tomato cultivation	field	positive effect on the uptake of important macro- and microelements, promotion of growth, increase in crop size	Allaga et al. 2020
*Trichoderma ghanense*, *T. afroharzianum*, *B. velezensis*, *P. resinovorans*	biological soil inoculant in sweet potato cultivation	field	improvement in plant tolerance to abiotic stress, promotion of growth in sweet potato plants, higher storage root yield	Nagy et al. 2023

IAA: indole acetic acid; AMF: arbuscular mycorrhizal fungus; SPAD: soil plant analysis development

The synergistic effect that occurs when *Trichoderma* species are used together can amplify the positive effects of the individual strains (Table 1). In the case of the co-application of strains *T. atroviride* and *T. citrinoviride*, compared with the single use of the strains, higher indole acetic acid (IAA) and iron-chelating siderophore content was measured, and synergism was assumed to have occurred between the species (Chen et al. 2021). They can produce higher amino acid and γ-aminobutyric acid content, which can provide further improvement of plant growth (Hao et al. 2022). The application of several *Trichoderma* isolates showed a significant effect against *Fusarium* wilt in bananas, while increased IAA production and phosphate solubilization were detected (Thangavelu and Gopi 2015). The inoculation of the conidial suspension from indole-producing *T. tomentosum* and *T. harzianum* strains, supplemented with the IAA precursor tryptophan, resulted in increased plant parameters, such as plant height, root length, leaf area and dry weights in maize (Herrera-Jiménez et al. 2018). The combined use of several *Trichoderma* species can provide wider protection against plant pathogens, while having a stronger supporting effect on the plants through the activation of immune response pathways (Bisen et al. 2019). The application of combined *Trichoderma* species on thiophanate-methyl coated dry bean seeds resulted in an increase in plant development and protection against *Fusarium solani* and *F. oxysporum* compared with the use of the individual strains (Abd-El-Khair et al. 2019). Properly selected species may have a significant impact not only in supporting plant growth, but also in mitigating the impact of environmental stress conditions, such as salt stress in rice production (Anshu et al. 2022).

### Combination of *Trichoderma* with other fungi

The critical features of *Trichoderma* species, the mycoparasitic and mycotrophic activities on the fungal targets, including taxonomically close species, may significantly affect and limit their combined use with other biocontrol fungi (Table 1).

He et al. (2022) recently found that co-inoculating plants with *T. viride* and dark septate endophytes creates a synergistic effect, enhancing the rhizosphere microbiome and the plants’ ability to adapt to drought, demonstrating *Trichoderma*’s compatibility with these endophytes.

The compatibility with mycorrhizal fungi, also considered indirect biocontrol agents, has been a debated issue due to their overlapping, competing activities for colonization sites, nutrients, and activating the systemic defenses in plants. There has been evidence about the mycoparasitic activities of *Trichoderma* on arbuscular mycorrhizal fungi (AMF) (Rousseau et al. 1996), and recent data suggest that by competing with *Glomus* spp., *T. harzianum* could significantly diminish the efficient disease-controlling impact of the AMF fungi in reducing the *Fusarium* wilt infection in bananas (Castillo et al. 2019).

In contrast, however, recent experiments when *Trichoderma* and mycorrhizal fungi were combined resulted in apparent compatibility and synergistic outcomes in improving disease resistance in tomatoes (Minchev et al. 2021) and promoting AMF-associated, improved plant productivity in a non-mycorrhizal *Brassica* host (Poveda et al. 2019). In tomato cultivation, strains of *T. harzianum* and *G. intraradices* have been used against Fusarium crown and root rot and have successfully inhibited the incidence and severity of the disease (Datnoff et al. 1995). The strains were also effective individually and in combination. In Nigerian cowpea seed and soil treatments, co-inoculation of *Glomus versiforme* and *T. harzianum* enhanced the resistance of cowpea seedlings against powdery mildew disease caused by *Erysiphe flexuosa* and also enhanced the growth of cowpea seedlings (Omomowo et al. 2018). The maximum increases in plant height, shoot fresh weight, root dry weight, number of leaves, root length and leaf area could be achieved with a *G. versiforme* mutant in combination with *T. harzianum*. *Trichoderma* spp. and mycorrhizal fungi were also examined in apple (Jonagold) tree nursery under replantation conditions. The growth of the root system and the aerial parts of the trees (including leaves) was much better after the combined use of both types of fungi than in the replant soil that had not received the fungal treatment. Both fungi had a good antagonistic effect on apple replant disease (ARD) (Zydl et al. 2021). Based on the above data, the compatibilities between various *Trichoderma* and AMF species must be tested case by case under both in vivo and in vitro conditions.

*Beauveria bassiana* and *T. asperellum* can synergistically suppress the immune response of the Asian corn borer, *Ostrinia furnacalis* and can be used as a sustainable approach to induce plant resistance through the activation of defence-related enzymes (Batool et al. 2020). The binary combination of *B. bassiana* and *T. asperellum* may enhance the lethal effect of *T. asperellum*. The seed coating method has been shown to be the most effective in terms of endophytic colonization of plants and may help in good plant growth. Using transcriptome analysis it was hypothesized that the expression of immunity-related genes was activated only when *T. asperellum* was inoculated, but expression was low in the case of *B. bassiana* treatment and the combined treatment, suggesting that the fungi are able to suppress the immune response of *O. furnacalis*. The use of these biopesticides can therefore be an environmentally friendly and sustainable approach to control insects, increase crop yields and eliminate the use of hazardous chemical pesticides (Batool et al. 2020).

### Combination of *Trichoderma* with bacteria

*Trichoderma* species can also be combined with beneficial bacteria with the aim that they complement each other (Table 1). In order to reach their full potential, the compatibility of the agents must be studied extensively (Triveni et al. 2012). Secondary metabolites produced by *Trichoderma* species are capable of strongly repressing bacteria present in the rhizosphere, so the bacterial communities might undergo significant changes during the treatment. On the other hand, the bacteria present in the rhizosphere might produce antifungal or other compounds that affect the efficiency of *Trichoderma* species as bioeffectors (Li et al. 2019).

Research on leveraging the benefits of combining plant growth-promoting bacteria with *Trichoderma* species is growing rapidly (Morales-Garcia et al. 2019; Table 1). These works focus on several crops including chickpea (Velmourougane et al. 2017), bean (Negi et al. 2019), wheat and maize (Karuppiah et al. 2019a, b; El-Katatny and Idres 2014), as well as tomato (Tomer et al. 2015) among other important agricultural plants (Poveda and Eugui 2022). The use of carriers proved to be also an important factor in the application of treatments, as they might influence synergistic effects. Neem cake and jatrofa cake affected the viability and performance of *T. harzianum* and *P. fluorescens*. (Tomer et al. 2015). The varieties of plant species also influence the results in a significant manner.

From a biocontrol perspective, several *Trichoderma* species have the ability to enhance protection against common plant diseases in combination with beneficial bacteria (Zhou et al. 2021). During the past two decades, isolates of over 10 *Trichoderma* species were investigated for their biocontrol properties in combination with several bacteria including *B. subtilis* (Kumar et al. 2015; Wang et al. 2019), *B. amyloliquefaciens* (Chien and Huang 2020; Wu et al. 2018), *P. fluorescens* (Madhavi et al. 2018), and *Rhizobium leguminosarum* (Saber et al. 2009), with promising results. The most common modes of action are induction of plant systemic (or local) resistance, production of secondary metabolites and lytic enzymes, competition for space, root colonization, parasitism, and increasing the number of beneficial microorganisms in the rhizosphere (Poveda and Eugui 2022).

Filtrates of *Trichoderma* species and four bacterial strains were tested in combinations via in vitro dual culture assays to determine their compatibility against white root rot in avocado plants caused by *Rosellinia necatrix* (Ruano-Rosa et al. 2014). The study found that combining *T. atroviride* with *Pseudomonas chlororaphis* and *Pseudomonas pseudoalcaligenes* strains was more effective than single treatments, delaying symptoms and reducing disease severity.

Recently, Liu et al. (2022c) conducted an experiment with *T. atroviride* SG3403 and *Bacillus subtilis* 22 applied as dry-powder seed coatings for the biocontrol of wheat head blight (*Fusarium gramineum*). Field experiments were also carried out in areas where the disease had been present for years, causing severe losses. The findings demonstrated that *T. atroviride* SG3403 is compatible with *B. subtilis* 22, and the presence of these microorganisms not only inhibited pathogen growth, but also reduced the production of the harmful mycotoxins deoxynivalenol and zearalenone (Liu et al. 2022c).

Leal et al. (2023) investigated the biocontrol of grapevine trunk diseases, caused by a complex group of pathogens resulting in huge economical losses. Experiments were carried out in nurseries with *T. atroviride* (Ta) SC1 and *B. subtilis* (Bs) PTA-271 in single and combined treatments. According to the results, combined treatments led to a reduction of disease incidence. The authors pointed out that more experiments are needed, because the environmental factors influence greatly the effectiveness of the treatment, and the follow-up evaluation of treated plants in the vineyard might also be necessary after planting (Leal et al. 2023).

It is important to note that based on several experiments, microorganisms in consortia might offer biocontrol and plant growth promoting effects simultaneously, without harming the native microbiome. Zhou et al. (2021) investigated *T. virens* Tvien6 and *Bacillus velezensis* X5 as biocontrol agents on tomato plants against bacterial wilt disease (*Ralstonia solanacearum*). Their results showed, that these agents also improved chlorophyll production, resulting in plant growth promotion. Petcu et al. (2023) reported an on-field maize study showing that a *T. harzianum* and *Bacillus subtilis* combination not only preserved beneficial soil organisms and crop quality but also enhanced plant growth and yield over two years. Treatments also reduced the incidence of maize leaf weevil (*Tanymecus dillaticolis*). Results were obtained after changing from classical to organic fertilizers, thus improving sustainability, underlining the importance of new farming practices (Petcu et al. 2023).

### Consortia with *Trichoderma* components

While the previously mentioned combinations typically involve two microbial strains, assembling consortia or synthetic communities could offer a more effective strategy to achieve a broader spectrum of beneficial effects (Table 1).

Yadav et al. (2013) carried out a study to evaluate the performance of three rhizosphere-competent microbial strains, namely *Pseudomonas fluorescens* OKC, *T. asperellum* T42 and *Rhizobium* sp. RH4, individually and in combination, in bioprimed seeds of chickpea and radish. Seeds were sown in pots and fields, and bioprimed seeds showed higher germination percentage and better plant growth in both crops compared to non-bioprimed control plants. All the combinations containing *Trichoderma* showed better results compared to the others and the triple microbial combination showed the best results in terms of germination and seedling growth in both chickpea and radish.

Allaga et al. (2020) developed a composite soil bioinoculant containing beneficial bacteria and fungi for biological control of plant pathogens, phosphorus mobilisation, stem decomposition, humification and nitrogen fixation. An isolate of *T. asperellum*, which has excellent in vitro antagonistic capabilities against several plant pathogenic fungi, was included as a potential biocontrol component. The selected strain also promoted the growth and photosynthetic activity of tomato plants. A *T. atrobrunneum* strain was selected for phosphorus mobilization and degradation of stem residues, which produced cellulose-degrading enzymes in the absence of stem residues, while this ability increased 10-15-fold in the presence of ground maize stover. The strain has also been shown to produce high levels of organophosphorus-releasing enzymes and cellulase and xylanase activities during solid-state fermentation on various plant residues. A *Streptomyces albus* strain with excellent peroxidase production capacity was selected as a potential humus-producing component, while an *Azotobacter vinelandii* strain capable of providing excess nitrogen to plants was used for nitrogen fixation. The soil bioinoculant had a positive effect on the uptake of some important macro- and microelements (potassium, sodium and manganese) from soil by field-grown tomato plants. The applied screening strategy has been shown to be applicable for the assembly of a composite soil bioinoculant with remarkable application potential (Allaga et al. 2020).

More recently, Nagy et al. (2023) established a microbial consortium consisting of two *Trichderma* strains (*T. ghanense* SZMC 25217, *T*. *afroharzianum* SZMC 25231) and three bacteria (*B. velezensis* SZMC 24986, *Arthrobacter globiformis* SZMC 25081, *P. resinovorans* SZMC 25872), selected for the biological control of plant pathogens, promotion of plant growth by phosphorus mobilisation and nitrogen supply, and the degradation of polysaccharides. Field trials in sweet potato with soil grafting showed that treated plants had higher average tuber size and yield per plant compared to untreated controls with and without fertilization. In the treatment type where sweet potato propagules were soaked and then post-inoculated, average tuber size and yield per plant were significantly higher than in the untreated control. The results led to the development of a microbial soil inoculant for sweet potato cultivation. Yield growth data suggested that the use of microbial mixtures of bacterial and fungal components is a promising approach to the efficient biological production of sweet potato (Nagy et al. 2023).

An intriguing prospect for the future is the potential for rhizospheric microbiome transplantation, where a whole microbiome is being examined, isolated and relocated to new habitats. While this method faces serious challenges, it provides plausible opportunities in sustainable agriculture (Orozco-Mosqueda et al. 2023).

### Conclusions and future prospects

**Fig. 1:**
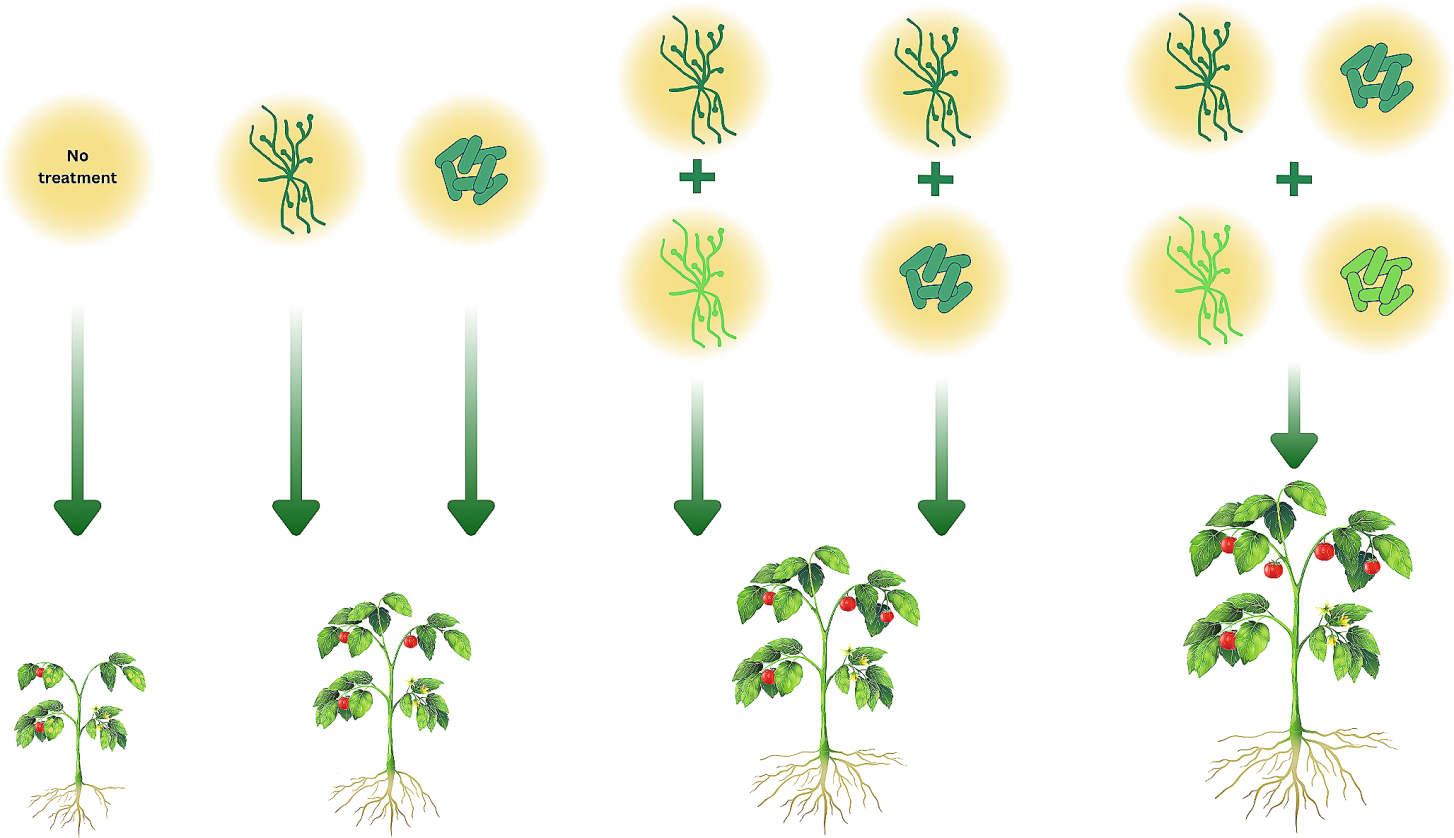
Effect of microbial inoculants on target plants. The combination of properly selected bioinoculant strains (*Trichoderma* with another *Trichoderma* or another fungus, *Trichoderma* with a bacterium, multiple *Trichoderma* with multiple bacteria) may have an additive or synergistic positive effect on the target plant

The combined use of microorganisms as biostimulants and biopesticides holds great potential for the future. Synergies between *Trichoderma* strains and other microorganisms may cause more benefits than the sum of their parts (Fig. 1), making them a promising alternative for crop management, disease and pest control, as well as crop plant stimulation in modern agriculture. However, further investigations are needed to determine the molecular background of the specific mechanisms behind the synergistic effects. Current research is focusing on using omics-based approaches to design biocontrol strategies, selecting effective and reliable microorganisms, and testing their combinations. Advances in metatranscriptomic and metabolomic analysis and other important molecular tools will provide insights in the interaction network to help researchers in reaching the full biocontrol and plant growth promoting potential of *Trichoderma* species and other plant-beneficial microorganisms. Recent findings regarding the upregulation of apoptotic genes in the target organism may offer a promising monitoring option to follow up the survival and contribution of various components of the microbial consortium (Chen et al. 2023). Multi-RNA-seq profiling of the apoptotic genes and the followup expression analysis of gene families significantly contributing to the biocontrol impact might be a highly informative source to identify and optimize the necessary partakers for the biocontrol intervention.

## Data Availability

No datasets were generated or analysed during the current study.
